# Associations between levels of oxidative nucleoside damage and cardiovascular risk in patients newly diagnosed with bipolar disorder and their unaffected relatives

**DOI:** 10.1038/s41398-022-02095-6

**Published:** 2022-08-10

**Authors:** Helena Lykke Bøgh, Sharleny Stanislaus, Hanne Lie Kjærstad, Kimie Stefanie Ormstrup Sletved, Julie Lyng Forman, Henrik Enghusen Poulsen, Maj Vinberg, Lars Vedel Kessing, Klara Coello

**Affiliations:** 1 Copenhagen Affective Disorders Research Centre (CADIC), Psychiatric Center Copenhagen, Copenhagen University Hospital Rigshospitalet, Copenhagen, Denmark; 2 Department of Clinical Medicine, University of Copenhagen, Copenhagen, Denmark; 3 Section of Biostatistics, Department of Public Health, University of Copenhagen, Copenhagen, Denmark; 4 Department of Endocrinology, Copenhagen University Hospital Bispebjerg Frederiksberg, Frederiksberg, Denmark; 5 Department of Cardiology, Copenhagen University Hospital North Zealand Hillerød, Hillerød, Denmark; 6 Research Unit, North Zealand Hospital Hillerød, Hillerød, Denmark; 7 Psychiatric Research Unit, Psychiatric Centre North Zealand, Copenhagen University Hospital, Hillerød, Denmark

**Keywords:** Prognostic markers, Bipolar disorder

## Abstract

Enhanced oxidative stress-generated nucleoside damage may contribute to the increased cardiovascular disease mortality in patients with bipolar disorder (BD) but the association has never been investigated. We investigated the associations between oxidative stress-generated damage to DNA (8-oxodG) and RNA (8-oxoGuo), respectively, and three measures reflecting cardiovascular risk; namely, the Framingham 30-year risk score of cardiovascular diseases, the metabolic syndrome, and the insulin resistance index in 360 patients newly diagnosed with BD, 102 of their unaffected relatives (UR) and 197 healthy control individuals (HC). In sex- and age-adjusted models, the 30-year cardiovascular risk score increased by 20.8% (CI = 7.4–35.9%, *p* = 0.002) for every one nM/mM creatinine increase in 8-oxoGuo and by 15.6% (95% CI = 5.8–26.4%, *p* = 0.001) for every one nM/mM creatinine increase in 8-oxodG, respectively. Further, insulin resistance index increased by 24.1% (95% CI = 6.7–43%, *p* = 0.005) when 8-oxoGuo increased one nM/mM creatinine. The associations between cardiovascular measures and oxidative nucleoside damage were more pronounced in patients with BD compared with UR, and HC. Metabolic syndrome was not associated with nucleoside damage. Overall, higher oxidative stress-generated nucleoside damage was associated with a higher cardiovascular risk score and a higher degree of insulin resistance index, and having BD impacted the associations. Further, within patients, treatment with psychotropics seemed to enhance the associations between 30-year CVD risk score and insulin resistance index, respectively, and oxidatively stress-generated nucleoside damage. Our findings support enhanced oxidative stress-generated nucleoside damage as a putative pathophysiological mechanism that may mediate the higher cardiovascular risk observed in patients with BD already at the time of diagnosis.

## Introduction

Oxidative stress contributes to accelerated ageing, which further seems to play key roles in the pathophysiology of both bipolar disorders (BD) [1–4], diabetes type 2 [5], and cardiovascular disease (CVD) [5, 6]. Further, enhanced oxidative stress is one of the putative pathophysiological mechanisms explaining the excessive CVD risk and mortality in patients with BD [7, 8]. Even in young age groups of patients with BD, CVD is the leading cause of physical comorbidity [9] and death [3, 8, 10] underpinning the importance of early intervention against traditional CVD risk factors from the onset of disease. Adolescents with BD seem to have lower levels of oxidative stress markers compared with adults with BD [11]. However, recently, in the Bipolar Illness Onset study (BIO study) [12], we found that systemic, oxidatively generated stress to DNA and RNA was also higher in patients newly diagnosed with BD as well as their unaffected first-degree relatives (UR) compared with healthy controls (HC) even when adjusting for lifestyle factors [13]. These findings align with other findings from our group of enhanced oxidative stress-generated nucleoside damage in patients with BD [14, 15]. In a large sample of elderly patients with type 2 diabetes (*n* = 1863), levels of oxidative stress-generated nucleoside damage predicted 5 years all-cause mortality as well as CVD mortality [5]. This association between oxidative stress levels and CVD risk has never been investigated in BD.

Metabolic syndrome (metS), a cluster of CVD risk factors including insulin resistance, is twice as prevalent in patients with BD compared with the general population and is associated with treatment resistance, a devastating illness trajectory, and premature death [16–18]. We have previously shown in the BIO study that patients newly diagnosed with BD are 3.5 times as likely as HC to fulfill the criteria of the metS and have a 20% higher insulin resistance index than HC, whereas UR neither differed from patients with BD nor from HC [19]. Overall, it is of interest to combine the findings of the three studies [13, 19, 20] based on the BIO sample hypothesizing that higher levels of oxidative stress are associated with a higher 30-year CVD risk score, higher insulin resistance, and higher prevalence of metS, respectively.

Aims of the study:

i. to investigate the association between oxidative stress-generated nucleoside damage and CVD risk, metS, and insulin resistance, respectively, across the entire sample without distinguishing between the study participant group,
ii. to investigate whether associations between oxidative stress-generated nucleoside damage and CVD risk, metS, and insulin resistance, respectively, differ between patients newly diagnosed with BD, their UR, and HC,
iii. to examine the effect of illness and medication variables on the association between oxidative stress-generated nucleoside damage and CVD risk in patients newly diagnosed with BD.

## Materials and methods

### Study design

The present study is a subset investigation presenting baseline data of the complete sample of participants in the ongoing longitudinal BIO Study, which aims to identify composite biomarkers for BD in patients newly diagnosed with BD, their UR, and HC [12]. The participants were included between June 2015 and April 2020.

The study protocol has been approved by the Committee on Health Research Ethics of the Capital Region of Denmark (protocol No. H-7-2014-007) and the Danish Data Protection Agency, Capital Region of Copenhagen (RHP-2015-023). The study complies with the Declaration of Helsinki and its ethical principles.

### Participants

#### Patients newly diagnosed with BD

Patients were recruited from the Copenhagen Affective Disorder Clinic. The Copenhagen Affective Disorder Clinic receives patients from the entire Capital Region of Denmark covering a catchment area of 1.6 million people as well as all psychiatric centers in the region, offering specialized treatment for patients with newly diagnosed/first-episode BD. All patients with newly diagnosed/first-episode BD were routinely invited to participate in the BIO study. Inclusion criteria were an International Classifications of Diseases, Tenth Revision (ICD-10) diagnosis of BD or a single manic episode, and an age of 15–70 years. Patients were excluded if BD occurred secondary to brain injury. The patients received treatment as usual while participating in this observational cohort study.

#### Unaffected first-degree relatives

With consent from included patients with BD, their unaffected first-degree relatives (UR) aged 15–70 (i.e., siblings and/or children) were invited to participate in the study. Relatives diagnosed with an ICD-10 psychiatric disorder in the psychotic or bipolar spectrum (i.e., ICD-10 F20–F31) were excluded from our study.

#### Healthy control individuals

Age- and sex-matched HC individuals, aged 15–70, without a personal or a first-degree family history of psychiatric disorders that had required treatment, were recruited on random days among blood donors from the Danish Blood Bank at Rigshospitalet, Copenhagen, Denmark, covering the same catchment area as The Copenhagen Affective Disorder Clinic.

### Diagnostics, data collection, and clinical assessment

An initial diagnosis was made by medical doctors specialized in psychiatry according to the ICD-10 and Diagnostic and Statistical Manual of Mental Disorder (DSM-IV) criteria for type I and type II BD. After informed written consent, all participants underwent a thoroughly assessment by medicine or psychology Ph.D. students verifying the diagnosis/lack of diagnosis using the Schedules for Clinical Assessment in Neuropsychiatry (SCAN) [21] categorizing patients as BD type I or type II and assessing comorbid psychiatric illness. Clinical assessments of the severity of depressive and manic symptoms during the preceding three days were done using the Hamilton Depression Scale-17 items (HAMD-17) [22] and the Young Mania Rating Scale (YMRS) [23]. Careful clinical evaluation was made including records of smoking habits, medication use, educational level, weekly alcohol intake, and objective measures of height, weight, and waist circumference as well as an assessment of physical activity level during the previous week using the International Physical Activity Questionnaire (IPAQ) [24]. Finally, blood pressure was measured using a calibrated automatic sphygmomanometer (Microlife BP A3 plus) in a sitting position after a 10-minute rest.

### Determination of the cardiovascular risk

We used three measures related to CVD risk. First, we investigated the Framingham 30-year hard risk score predicting coronary death, myocardial infarction, and stroke in patients between the ages of 20–59 without preexisting CVD and cancer [25]. The 30-year Framingham CVD risk score tool is validated from 20 years of age, and long-term risk prediction seems to forecast overt and subclinical CVD more accurately in a young population compared with scores based on relatively shorter duration [25–27]. Second, metS was investigated using the International Diabetes Federation’s definition [16] and third, we assessed the insulin resistance index, which is an individual risk factor of type 2 diabetes and CVD [25]. Further details of our applied CVD risk assessments are described in greater detail elsewhere [19, 20].

### Laboratory methods

Blood samples were collected in a fasting state between 7.30 a.m. and 10 a.m. on the day of clinical assessments, after participants had a period of minimum 15-minute rest. Five milliliters of blood were drawn by venipuncture into an EDTA-containing vacuum tube (Vacuette®) and within 30 min centrifuged at 1590 × *g* and 4 °C for 15 min.

Plasma/serum concentrations of glucose, Hemoglobin 1Ac (HbA1c), high-density lipoprotein (HDL), triglyceride, and cholesterol were measured as part of the standard laboratory routine, whereas insulin was measured in a subpopulation of *n* = 336 after storage at −80 °C (191 patients with BD, 45 UR, and 100 HC). At the Department of Clinical Biochemistry, Rigshospitalet, Copenhagen, Denmark, educated technicians, blinded with respect to participant status, collected blood samples, and handled all aspects of laboratory processing. For further details on laboratory methods please see [19, 20].

A freshly voided spot urine sample was obtained using a standard sampling kit without any additives and collected in the morning at the day of clinical assessment (In Vitro, Fredensborg, Denmark) and aliquoted into Eppendorf® tubes and kept frozen at −80 °C until levels of 8-oxo-7,8-dihydro-2’-deoxyguanosine (8-oxodG), 8-oxo-7,8-dihydroguanosine (8-oxoGuo) were assayed.

Urine samples were analyzed using ultra-performance liquid chromatography and tandem mass spectrometry (UPLC-MS/MS) in the Laboratory of Clinical Pharmacology, Rigshospitalet, Copenhagen, Denmark, for further details please see [28]. Creatinine concentrations were measured in the urine samples for oxidative stress levels to be divided by creatinine levels in accordance with Jaffe’s reaction to adjust for the glomerular filtration rate [29].

### Statistical analyses

Categorical data were summarized in numbers and percentages and continuous data in medians and quartiles.

The apparent association of the 30-year Framingham CVD risk score and the insulin resistance index, respectively, with 8-oxodG and 8-oxoGuo, respectively, was investigated in a linear mixed effect model with the familial relationship as a random effect to account for the correlation between family-related individuals. Both outcomes had a skew distribution and were log-transformed, hence normal distribution was obtained, prior to analysis. Hence results are reported as a relative increase per one nM/mM creatinine increase in 8-oxodG and 8-oxoGuo with 95% confidence intervals. Similarly, the association of metS with 8-oxodG and 8-oxoGuo was analyzed in a generalized linear mixed effect model and reported with odds ratios (OR) and 95% confidence intervals.

In our **primary analyses,** the associations between nucleoside damage and cardiovascular risk were evaluated while further adjusting for sex and age in the mixed effects models. We controlled for false discovery rates by adjusting for multiple testing using the FDR-controlling procedure. In our **secondary analyses**, we compared the study participant group (BD, UR, and HC) by further including this and its interaction with 8-oxodG and 8-oxoGuo, respectively. Results from primary and secondary analyses were illustrated in forest plots.

Exploratory subgroup analyses were made for patients newly diagnosed with BD according to their HAMD-17 total score (<14 versus ≥14), YMRS total score (<14 versus ≥14), illness duration (<5 versus ≥5 years), and a number of affective episodes (<5 versus ≥5), receiving lithium (yes/no), antiepileptics (yes/no), antidepressants (yes/no) and antipsychotics (yes/no), as well as psychotropic medication (yes/no). All analyses were adjusted for sex and age. Subgroup analyses were only performed when the subgroup included at least 30 cases. Results were illustrated in forest plots.

Statistical analyses were performed using SPSS version 25 and R version 4.1.0.

## Results

We included 360 patients with BD, 102 UR, and 197 HC, see Table 1. Of the patients, 97.2%, were diagnosed with BD within the last two years. All three groups had a BMI and waistline circumference within the normal range; however, patients with BD had higher BMI compared with HC. The prevalence of current smoking in patients with BD was higher compared with HC and UR, whereas, physical activity level (IPAQ) did not differ between groups, see Table 1. Among patients with BD, the median illness duration since the first depressive, (hypo)manic, or mixed episode was 10 years and the median duration of untreated BD, defined as the time from the first manic, hypomanic, or mixed episode, was four years, see Table 1. Most patients were in full or partial remission based on ICD-10 criteria at the baseline visit. Seven of the UR had previously had a depressive episode that had required treatment and one UR fulfilled the criteria for alcohol dependence.

**Table 1:** Demographic and clinical variables in patients with newly diagnosed bipolar disorder (BD), their unaffected relatives (UR), and healthy controls individuals (HC).

	BD	UR	HC
*N*	360	102	197
Age (years)	29.0 [24.2–36.9]	26.7 [22.8–32.3]	27.6 [24.3–36.1]
Sex (% female)	234 (65.0)	59 (57.8)	127 (64.5)
Education (years total)	15 (13–17)	15 (13–17)	16 (15–17)
BMI (kg/m^2^)	24.5 (22–27)	23.8 (21–27)	23.7 (22–26)
Waist circumference (cm)	85 [77–93]	82 [75–91]	81 [74–89]
Number of smokers (%)	156 (43.9)	22 (22.0)	22 (11.2)
Alcohol (units per week)	2 [0–7]	2 (1–6)	5 (2–10)
IPAQ	1983 [1040–3685]	2400 [1085–4773]	2799 [1538–4262]
HAMD-17	9 (5–15)	2 [0–4]	0 [0–2]
YMRS	3 [0–7]	0 [0–2]	0 [0–1]
BD I	112 (31.1)	—	—
BD II	248 (68.9)		
Age of onset (years)	17 (14–21)	—	—
*Illness duration (years)	10 (6–16)	—	—
**Untreated bipolar disorder (years)	4 (1–10)	—	—
Affective episodes	12.5 (6–27)	—	—
Current state			
Remission	211 (58.9)	—	—
Mild/moderate depressive episode	86 (24)	—	—
Severe depressive episode	8 (2.3)	—	—
Manic episode	1 (0.3)		
Hypomanic episode	30 (8.4)	—	—
Mixed episode	20 (5.6)	—	—
N/A	2 (0.6)	—	—
Psychotropics			
No psychotropic medication	61 (16.9)	—	—
Antidepressant treatment	47 (13.1)	—	—
Antipsychotic treatment	120 (33.3)	—	—
Antiepileptic treatment	187 (51.9)	—	—
Lithium treatment	110 (30.6)	—	—
UR with a psychiatric disorder	—	8 (7.8)	—
Absolute CVD risk (%), *n* = 604	3.38 [1.5–7.6]	2.27 [1.1–5.2]	2.07 [1.2–4.9]
MetS, *n* = 612	49 (13.9)	10 (10)	13 (6.6)
Insulin resistance index, *n* = 336	2.06 [1.5–3.3]	2.06 [1.5–3.3]	1.73 [1.3–2.4]

Continuous variables are presented as median [interquartile range]. Categorical variables are presented as *n* (%).

*BMI* body mass index, *IPAQ* International Physical Activity Questionnaires, HAMD-17 Hamilton Depression Rating Scale, *YMRS* Young Mania Rating Scale, *N/A* not applicable, *CVD* cardiovascular disease, *Mets* metabolic syndrome.

*Illness duration was defined as the time from first episode (i.e., depressive, manic, hypomanic, or mixed episode).

**Untreated bipolar disorder was defined as time from first (hypo)manic or mixed episode to the time of diagnosis.

The 30-year CVD risk score was higher in patients with BD than in UR and HC, respectively, see Table 1. The insulin resistance index and prevalence of metS were also higher in patients with BD compared with HC, whereas neither metS nor insulin resistance index differed substantially between UR and patients with BD or between UR and HC, see Table 1.

### Associations between oxidative stress and CVD risk measurements in the complete study population

**Fig. 1:**
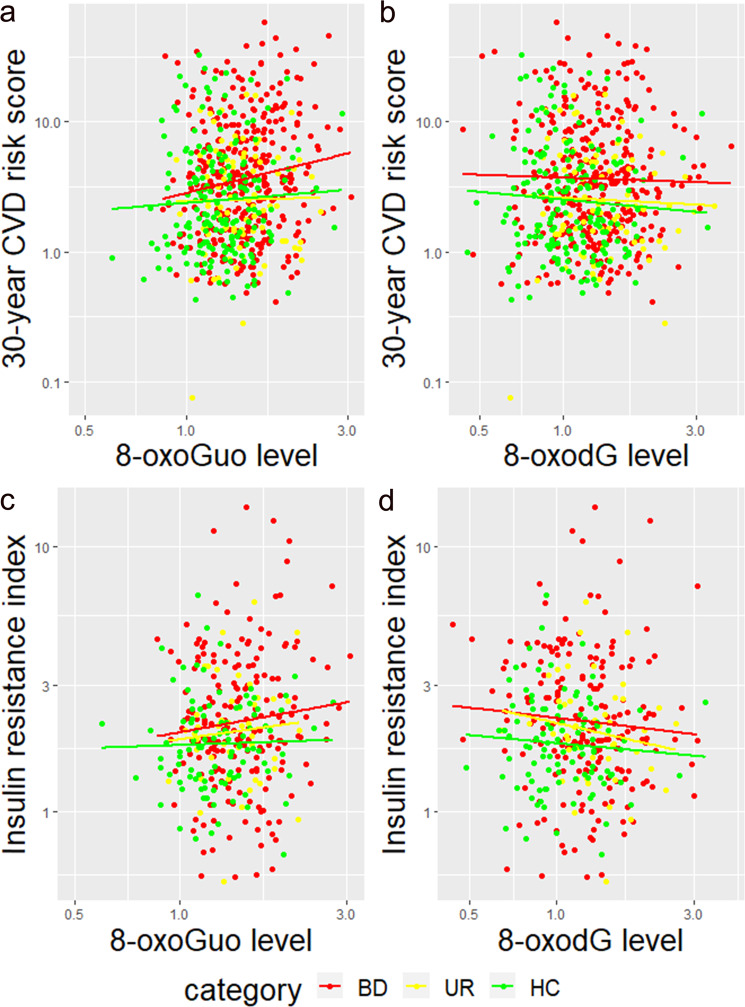
Associations between cardiovascular disease risk measurements and oxidative stress in the complete study population. Scatter plot of the 30-year cardiovascular disease risk score against levels of 8-oxoGuo (**a**) and 8-oxodG (**b**) and insulin resistance index against levels of 8-oxoGuo (**c**) and 8-oxodG (**d**). Fit lines are drawn for the three subgroups individually. BD bipolar disorder, UR unaffected relatives, HC healthy control individuals. Outcome variables were transformed by the natural logarithm.

In unadjusted analyses, one nM/mM creatinine increase in 8-oxoGuo was associated with 48.7% higher 30-year CVD risk score and 20.4% higher insulin resistance index, respectively, whereas no associations with 8-oxodG were found, see Table 2**Model 1** and Fig. 1. The association between higher levels of 8-oxoGuo and higher CVD risk score as well as higher insulin resistance survived FDR correction for multiple testing, see Table 2 Model 1. No statistically significant associations between metS and nucleoside damage were found.

**Table 2:** The association between 30-year cardiovascular risk score, metabolic syndrome, and insulin resistance index (dependent variables), respectively, and oxidative stress markers 8-oxoGuo and 8-oxodG (independent variables), respectively.

	Model 1: unadjusted				Model 2: adjusted for sex and age			
	*B*	95% CI	*P*	*P*_adj_	*B*	95% CI	*P*	*P*_adj_
30-year CVD risk score								
8-oxodG	0.1	−15.6–118.8	1.0	1	15.6	5.8–26.4	0.001	0.005
8-oxoGuo	48.7	19.1–85.5	<0.001	0.003	20.8	7.4–35.9	0.002	0.005
Metabolic syndrome								
8-oxodG	0.14	0.008–2.3	0.2	0.3	0.20	0.011–3.5	0.3	0.4
8-oxoGuo	8.13	0.907–73	0.1	0.2	11	0.846–14.3	0.1	0.2
Insulin resistance index								
8-oxodG	−4.8	−16.1–8.0	0.4	0.5	−3.3	−14.8–9.8	0.6	0.6
8-oxoGuo	20.4	3.8–39.7	0.015	0.045	24.1	6.7–44.3	0.005	0.01

Model 1: unadjusted linear mixed-effects models (continuous outcomes) and generalized linear mixed effect regression models (dichotomous outcome) with familial factor as a random factor. Model 2: sex and age-adjusted linear mixed effects regression models (continuous outcomes) and generalized mixed effect regression models (dichotomous outcome) with familial factor as a random factor (primary analyses).

*B* back transformed beta values reflecting the % or increment in dependent variable according to one nm/nM creatinine increment in independent variable (8-oxodg or 8-oxoguo, respectively), *CI* confidence interval, *P*_adj_ FDR adjusted *P* value, *8-oxodG* 8-oxo-7,8-dihydro-2’-deoxyguanosine, *8-oxoGuo* 8-oxo-7,8-dihydroguanosine.

In the **primary analyses**, adjusted for sex and age, we found elevated 30-year CVD risk score increased 15.6% and 20.8%, respectively, with one increment in 8-oxodG and 8-oxoGuo, respectively, and these associations persisted when controlling for multiple testing, see Table 2**Model 2**. In similar models, the insulin resistance index levels increased 24.1% with an increment of one nM/mM creatinine of 8-oxoGuo, and this association persisted when correcting for multiple testing, whereas no association between insulin resistance index and 8-oxodG was found, see Table 2**Model 2**. The prevalence of metS was neither associated with 8-oxodG levels nor 8-oxoGuo levels, see Table 2**Model 2**.

### Associations between oxidative stress and CVD risk measurements within study participant groups

**Fig. 2:**
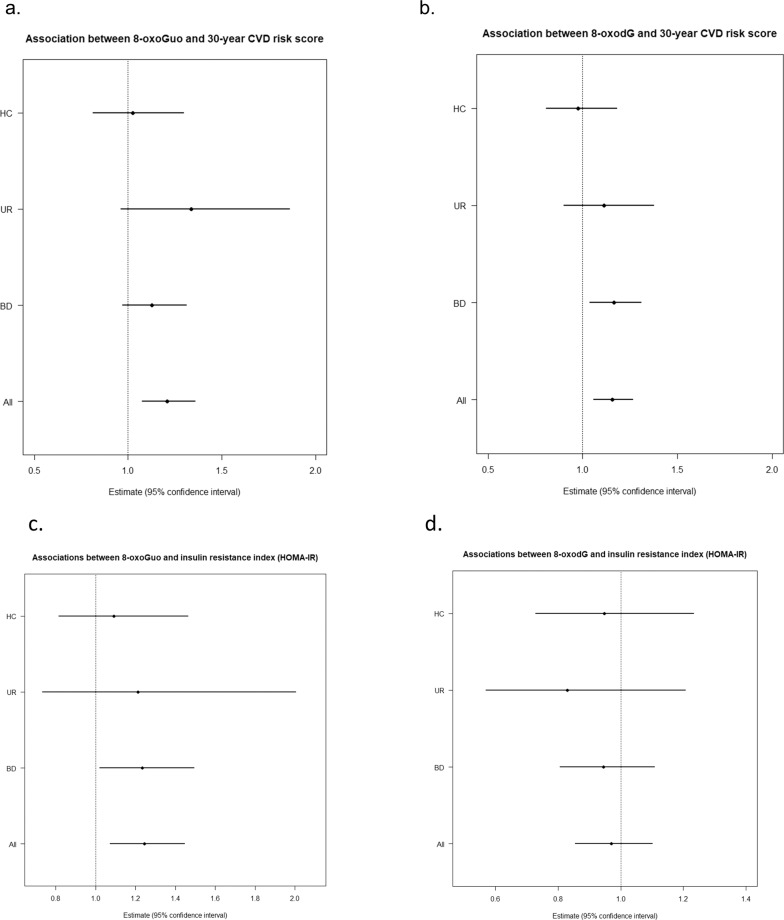
Associations between cardiovascular disease risk measurements and oxidative stress comparing participant groups. Forest plots of associations between 30-year cardiovascular disease risk score and levels of 8-oxoGuo (**a**) and 8-oxodG (**b**) and insulin resistance index and levels of 8-oxoGuo (**c**) and 8-oxodG (**d**), comparing total study population (“All”) with the three subgroups: BD patients with bipolar disorder; UR unaffected relatives, HC healthy control individuals.

In the **secondary analyses** adjusted for sex and age, we found an effect of BD on the association between 30-year CVD risk score and 8-oxodG, however, as shown in Fig. 2b confidence intervals were overlapping with that of UR and HC. The same pattern with an effect of BD was found on the association between insulin resistance index and oxoGuo (Fig. 2c). As depicted no effects of the study participant group were detected on the associations between 30-year CVD risk score and 8-oxoGuo (Fig. 2a) nor between insulin resistance index and levels of 8-oxodg8-oxodG (Fig. 2d). Due to limited cases of metS in UR and HC we did not have the power to run interaction analyses investigating the association between metS and oxidative stress-generated nucleoside damage.

To further investigate whether the patients with BD were driving the associations between 30-year CVD risk score and insulin resistance index, respectively, and nucleoside damage in the complete sample (primary analyses) in exploratory sensitivity analyses we reran these analyses this time only including UR and HC. In these analyses, the association between 30-year CVD risk score and levels of 8-oxoGuo (*B* = 12.9%, 95% CI = 9.46–34.8, *p* = 0.2, *p*_adjusted_ = 0.3) and 8-oxodG (30-year CVD risk score: *B* = 5.9%, 95% CI = −6.6–20.2, *p* = 0.4, *p*_adjusted_ = 0.5) attenuated and no longer reached statistical significance. Similarly, no association between insulin resistance index and 8-oxoGuo was found in analyses solely including UR and HC (*B* = 21%, 95% CI = 4–40, *p* = 0.3, *p*_adjusted_ = 0.4). As in our primary analyses, insulin resistance index and 8-oxodG levels were not associated when only including UR and HC (*B* = −9%, 95% CI = −22.4–7.0, *p* = 0.4, *p*_adjusted_ = 0.5).

### Association between oxidative stress and CVD risk measurements in patients newly diagnosed with BD

**Fig. 3:**
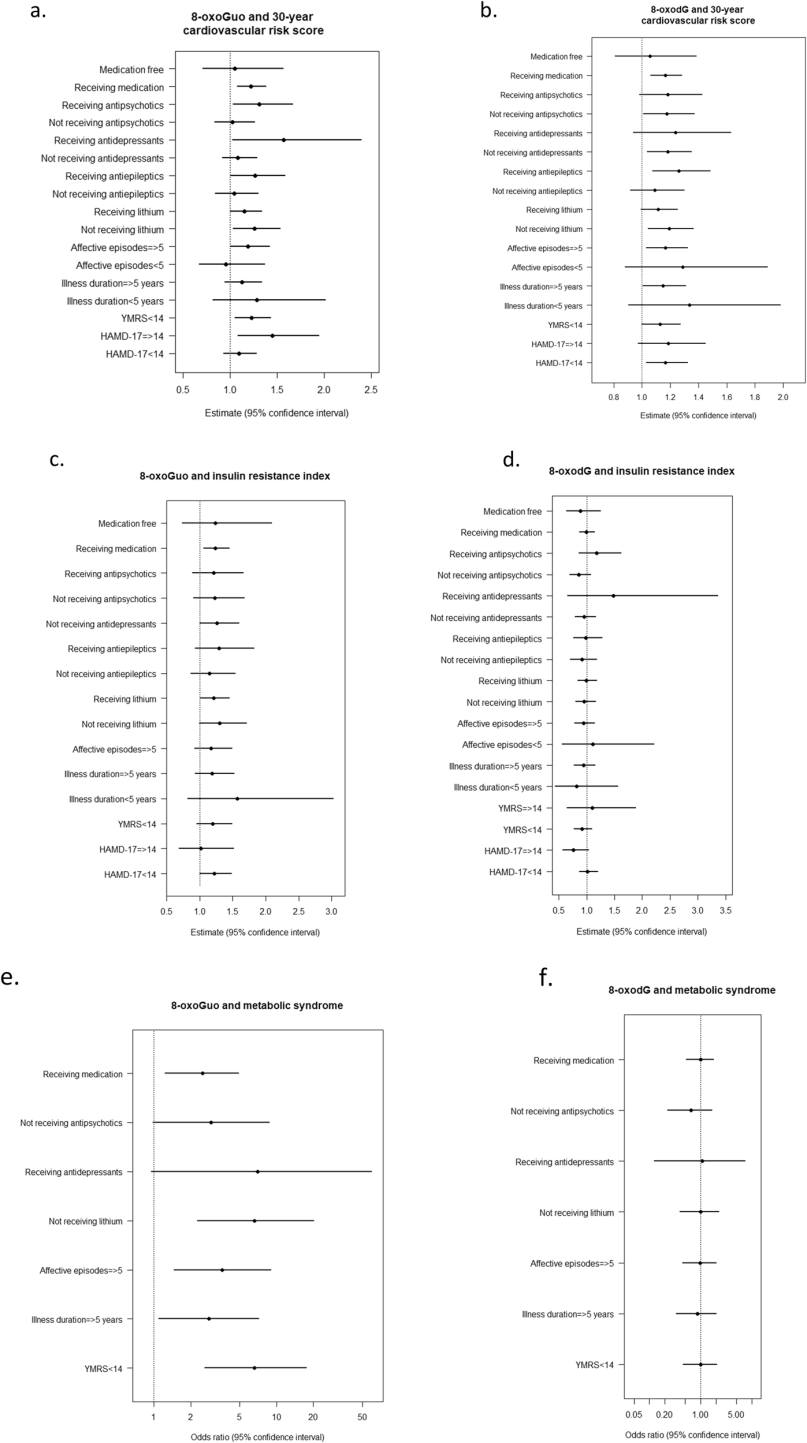
Associations between cardiovascular disease risk measurements and oxidative stress within patients newly diagnosed with bipolar disorder. Forest plots exploring the effects of illness- and medication variables on the associations between 30-year cardiovascular risk score and levels of 8-oxoGuo (**a**) and 8-oxodG (**b**), insulin resistance and levels of 8-oxoGuo (**c**) and 8-oxodG (**d**) and metabolic syndrome and levels of 8-oxoGuo (**e**) and 8-oxodg8-oxodG (**f**) in patients newly diagnosed with bipolar disorder. Analyses were run as separate multiple linear regression analyses adjusted for sex and age.

In separate regression analyses within patients with BD adjusted for sex and age, we found the association between 30-year CVD risk score and 8-oxoGuo was enhanced in subgroups with HAMD-17 score ≥14 or YMRS score <14, respectively (Fig. 3a), whereas the association between 30-year CVD risk score and 8-oxodG was not affected by symptom severity (Fig. 3b). Illness duration did not affect the associations, whereas higher number of affective episodes enhanced the association between 30-year CVD risk score and 8-oxodG (Fig. 3b). Receiving psychotropics seemed to influence the association between 30-year CVD risk score and levels of 8-oxoGuo and 8-oxodG, respectively (Fig. 3a, b). Further, receiving antiepileptics influenced the association between 30-year CVD risk score and 8-oxodG levels, whereas receiving antidepressants and receiving antipsychotics were both affecting the association between 30-year CVD risk score and 8-oxoGuo levels, see Fig. 3a, b. Finally, the subgroup of patients not receiving lithium had enhanced associations between 30-year CVD risk score and oxidative stress-generated nucleoside damage (Fig. 3a, b). Notably, lithium was received by 50% of patients with BD type I and 22% of patients with BD type II.

The only association between insulin resistance index and nucleoside damage identified, was receiving psychotropic medication, which had a small impact on the association between insulin resistance and 8-oxoGuo, see Fig. 3c, d.

The association between metS and 8-oxoGuo levels was enhanced in patients with BD receiving psychotropic medication, not receiving lithium, and having a YMRS score below 14, respectively. Further, the association was affected by increasing illness burden, measured as illness duration of more than five years and number of affective episodes more than five, see Fig. 3e. No effects of illness and medication variables were found on the associations between metS and 8-oxodG, see Fig. 3f.

## Discussion

### Main findings

This study profited from integrating validated measurements of systemic oxidative stress-generated nucleoside damage with well-established CVD risk measures in a large well-characterized study sample of 659 participants comprising 360 patients newly diagnosed with BD, 102 UR, and 197 HC. We found that higher oxidative stress-generated nucleoside damage was associated with higher 30-year CVD risk score and higher insulin resistance index. The associations between RNA damage and insulin resistance index as well as between DNA damage and 30-year CVD risk score were more pronounced in BD compared with UR and HC. Further, the investigated CVD risk measures were more prominent in patients with BD, but not in UR, compared with HC. The prevalence of metS was overall relatively low, and no associations between metS and oxidative stress were found.

Within patients, receiving psychotropic treatment correlated with enhanced associations between cardiovascular measures and levels of nucleoside damage.

### Interpretation of findings regarding 30-year cardiovascular risk score

As hypothesized, we found a positive association between higher levels of oxidative stress and higher 30-year CVD risk score across the full study population in this relatively young cohort (median age 28 [8, 23–34]), which is in line with previous findings from other patient populations [5, 6]. We found an effect of BD on association between oxidative stress-generated DNA damage and 30-year CVD risk score in line with a prior study, where adolescent patients with BD had a stronger association between peripheral oxidative stress markers and carotid-intima thickness, a proxy measure of CVD disease [30], compared with HC [11]. The excessive overlap between CVD and BD have been suggested to exceed the role of expected risk factors [7, 17, 31]. However, our findings only partly support this hypothesis, as we did find a stronger association between 30-year CVD risk score and DNA damage but not between 30-year CVD risk score and RNA damage and further the confidence intervals of the three participant groups were highly overlapping. Nonetheless, we found a tendency towards patients with BD and to a lesser extend their UR had a positive effect on the association between 30-year CVD risk score and oxidative stress-generated nucleoside damage compared with HC, and the largely overlapping confidence intervals could indicate that our sample was underpowered to detect true differences (type II errors). Further, we only focused on one of several suspected putative pathophysiological mechanisms leading to an increased CVD risk [8], and our findings cannot exclude patients with BD being prone to CVD in a degree that exceeds traditional risk factors. Further, in exploratory analyses only including HC and UR we did not find associations between 30-year CVD risk score and oxidative stress markers, suggesting that patients newly diagnosed with BD were largely driving these findings.

As previously found in a subsample of the present sample [20], patients newly diagnosed with BD had almost twice as high absolute CVD risk as HC (3.38% vs. 1.73%) and this risk was also statistically significantly higher than that of UR (2.02%). We expected to find higher 30-year CVD risk in UR compared with HC due to their higher levels of nucleoside damage [13] and higher 30-year CVD risk score found in a subsample of the present UR cohort compared with HC [20]. However, against expectations, in this sample including twice as many UR, UR did not differ from HC in 30-year CVD risk score. This finding comports with recent findings from a Danish nationwide population-based register study, where siblings of patients with BD, who were themselves unaffected by BD, did not have increased rates of CVD [9]. Notably, the younger age of UR, may explain that the higher oxidative stress levels were not translated into a higher 30-year CVD risk as age is a strong risk factor in the Framingham CVD risk calculator [25]. Further, the sample size of UR was smaller than intended due to recruitment difficulties, which resulted in rather broad confidence intervals as shown in Fig. 2. Thus, we may have overlooked a true association between 30-year CVD risk and oxidative stress in UR.

Our findings of higher 30-year CVD risk in patients with BD compared with UR and HC may partly be due to psychotropic medication as well as an unhealthier lifestyle. Noticeably, the prevalence of smoking, a major risk factor for CVD [6], differed substantially among groups with a prevalence of 43.9% among patients with BD versus 22% of UR and 11.2% of HC (Table 1). Further, patients newly diagnosed with BD had a higher BMI and wider waist circumference compared with UR and HC, whereas physical activity levels did not differ between groups (Table 1). Within patients we found an effect of psychotropic medication enhancing the association between 30-year CVD risk and oxidative stress-generated nucleoside damage, however, interestingly, not receiving lithium also enhanced the association supporting a cardiometabolic protective effect of lithium [32], which may possibly be mediated by reducing oxidative stress [33]. Nonetheless, cautious interpretation of these findings concerning lithium are warranted due to the difference in the prescription pattern of lithium to patients with BD type I versus II. Thus, in our cohort 50% of patients with BD type, I received lithium contrasting only 22% of patients with BD type II, and the latter subgroup was more than twice as large as the BD type I subgroup emphasizing that an impact of BD type on the found associations cannot be excluded.

Our findings comport with oxidative stress being a putative pathophysiological mechanism contributing to accelerated atherosclerosis and early CVD in adolescent and young adults with BD [8, 17]. The higher oxidative stress damage present in patients newly diagnosed with BD and UR underscores the likely mitochondrial dysfunction present already in the early stages of BD and possibly preceding BD [34–36] leading to a susceptibility to early CVD [37]. However, based on our study design we cannot determine causation.

### Interpretations of findings regarding metabolic syndrome and insulin resistance index

Metabolic syndrome was more prevalent in patients newly diagnosed with BD, but not in UR, compared with HC, in line with our prior findings in a subsample of the present sample [19]. Contrasting prior findings [38] no association between metS and oxidative stress was found in the present larger study sample. However, the overall relatively low prevalence of metS and relatively high OR and broad confidence intervals between metS and 8-oxoGuo could either indicate lack of power or lack of effect and results should be interpreted with caution.

Receiving psychotropic medications, not receiving lithium, and increasing illness burden impacted the association between metS and oxidative stress-generated nucleoside damage, however, we could not investigate several of the planned illness and medication variables due to few cases in subgroups. A low score on the YMRS corresponding to full og partial remission of manic symptoms also had an effect and we did not have the statistical power to investigate a possible effect of depressive symptoms. In the present study, the International Diabetes Federations’ definition of metS was used in which central obesity is measured as increased waist circumference and is a required criterion to fulfill the syndrome definition [16]. Most of the included participants were young adults and all three study participant groups had BMIs within the normal range and normal waistline circumferences; accordingly, the prevalence of metS was relatively low in the three groups (6.6–13.9%). However, insulin resistance index, a component of the more advanced assessment of metS [16], often preceding development of type 2 diabetes and CVD [39, 40], was positively associated with 8-oxoGuo across the three participant groups, however, this association did not persist in exploratory analyses only including UR and HC. Along this line, we found an enhancing effect of the BD participant group on the association between insulin resistance index and levels of 8-oxoGuo, however, confidence intervals between the three study participant groups were wide and largely overlapping, thus, interpretations should be made with caution. Within patients newly diagnosed with BD receiving psychotropics impacted the association between insulin resistance index and RNA damage, whereas no other effects were found of the investigated illness- or medication variables.

### Strengths and limitations

It is a strength that we included a large cohort of well-described patients newly diagnosed with BD, their UR, and HC. The study further profited from a high degree of methodological standardization with blood and urinary samples being collected in a fasting state between 7.30–10 A.M. Oxidative stress-generated nucleoside damage was measured using the golden standard analyses method [28] and all aspects of blood- and urine samples were analyzed and handled by experts in the field blinded to participant status. Biological samples were collected on the same day as participants were having a thorough clinical evaluation by a medical doctor or a psychologist trained in diagnosing BD.

Nonetheless, some limitations apply to the study. First, HC were recruited among sex- and age-matched blood donors from the same catchment area as patients with BD, whom had a higher level of education than patients with BD and UR. However, patients newly diagnosed with BD may have been delayed in their education due to their BD diagnosis [41], whereas the UR most likely had a lower educational level due to their younger age. Blood donors generally represent a rather healthy population possibly distorting the results by increasing the difference compared with patients with BD [30, 42]. Nonetheless, using a very healthy comparison group makes it easier to distinguish illness mechanisms and understand pathology, thus, we consider the control group a proper and pragmatic choice. Further, other ways to recruit HC such as via advertisements or national registers often result in low response rates and selection bias. Secondly, the 30-year Framingham risk score is validated in the age range 20–59 years accordingly we only included participants in this age range leading to exclusion of 53 study participants. However, we included metS and insulin resistance index as other CVD risk measurements, which were not age restricted. Third, the insulin resistance index was only measured in approximately half of the study population, nonetheless, the subpopulation is symmetrically represented by roughly half the number of participants in each group. Thus, we cannot rule out that we possibly had a statistical power issue that may have resulted in false-negative findings. Fourthly, we used dichotomous treatment categories, which fail to capture the effects of dose and duration of treatment or overlap of treatments. However, we repeated the post hoc analyses with the binary categorical value receiving medication (yes/no) and psychotropic medication was correlated with enhanced associations between cardiovascular measures and levels of nucleoside damage. Finally, confounding-by-indication should be considered as psychotropic medication was not prescribed randomly but selected by the psychiatrist based on the patient’s clinical features (e.g., BD type I and II), which may be associated with the outcome.

## Conclusion

Overall, higher oxidative stress levels were associated with a higher 30-year CVD risk score and a higher degree of insulin resistance index, and having BD impacted the associations. Further, within patients, treatment with psychotropics correlated with enhanced associations between cardiovascular measures and oxidative stress-generated nucleoside damage.

Our findings support enhanced oxidative stress-generated nucleoside damage as an important pathophysiological mechanism already present at the early stages of BD emphasizing the importance of an early and integrated treatment strategy including both psychiatric and physical health in patients with BD.
